# Model for human skin reconstructed *in vitro* composed of associated dermis and epidermis

**DOI:** 10.1590/S1516-31802006000200005

**Published:** 2006-03-02

**Authors:** Luís Ricardo Martinhão Souto, Jussara Rehder, José Vassallo, Maria Letícia Cintra, Maria Helena Stangler Kraemer, Maria Beatriz Puzzi

**Keywords:** Cell culture, Collagen, Fibroblasts, Keratinocytes, Skin, Cultura de células, Colágeno, Fibroblastos, Queratinócitos, Pele

## Abstract

**CONTEXT AND OBJECTIVE:**

The technique of obtaining human skin with dermis and epidermis reconstructed from cells isolated from patients can enable autologous skin grafting on patients with few donor sites. It also enables *in vitro* trials on chemicals and drugs. The objective of this work was to demonstrate a method for obtaining human skin composed of associated dermis and epidermis, reconstructed *in vitro*.

**DESIGN AND SETTING:**

Experimental laboratory study, in the Skin Cell Culture Laboratory of Faculdade de Ciências Médicas, Universidade Estadual de Campinas.

**METHODS:**

Cells from human fibroblast cultures are injected into bovine collagen type I matrix and kept immersed in specific culturing medium for fibroblasts. This enables human dermis reconstruction *in vitro*. On this, by culturing human keratinocytes and melanocytes, differentiated epidermis is formed, leading to the creation of human skin composed of associated dermis and epidermis, reconstructed *in vitro*.

**RESULTS:**

We showed that human skin composed of associated dermis and epidermis can be successfully reconstructed *in vitro*. It is histologically formed in the same way as human skin *in vivo*. Collagen tissue can be identified in the dermis, with cells and extracellular matrix organized in parallel to multilayer epidermis.

**CONCLUSIONS:**

It is possible to obtain completely differentiated human skin composed of associated dermis and epidermis, reconstructed *in vitro*, from injection of human fibroblasts into bovine collagen type I matrix and culturing of human keratinocytes and melanocytes on this matrix.

## RESUMO

**CONTEXTO E OBJETIVO:**

A técnica para obtenção de pele humana que apresente derme e epiderme, reconstruída a partir de células isoladas de pacientes, pode possibilitar a realização de enxertos autólogos de pele reconstruída em laboratório em pacientes com áreas doadoras escassas, além de permitir ensaios com substâncias químicas e drogas *in vitro* e não mais *in vivo*. O objetivo do trabalho é demonstrar um método de obtenção de pele humana reconstruída *in vitro* composta de derme e epiderme associadas.

**TIPO DE ESTUDO E LOCAL:**

Estudo experimental laboratorial realizado no Laboratório de Cultura de Células da Pele da Faculdade de Ciências Médicas da Universidade Estadual de Campinas, Campinas, São Paulo, Brasil.

**MÉTODOS:**

A partir da cultura de fibroblastos humanos, é possível obter um número suficiente de células que podem ser injetadas em uma matriz de colágeno bovino tipo I que, mantida imersa em meio de cultura específico para fibroblastos, permite a formação de uma derme humana reconstruída *in vitro*. Sobre essa derme, por meio de cultura de queratinócitos e melanócitos humanos, forma-se epiderme diferenciada, levando à formação de pele humana reconstruída *in vitro,* composta de derme e epiderme associadas.

**RESULTADOS:**

Demonstramos que é possível reproduzir pele humana reconstruída *in vitro,* composta de derme e epiderme associadas. Essa pele humana formada é, histologicamente, semelhante à pele humana *in vivo*. Na derme, identifica-se o tecido colágeno, com suas células, e a matriz extracelular organizados paralelamente à epiderme. Esta se desenvolve em várias camadas.

**CONCLUSÃO:**

É possível obter pele humana reconstruída *in vitro*, completamente diferenciada, composta de derme e epiderme, associadas, a partir da injeção de fibroblastos humanos em uma matriz de colágeno bovino tipo I e da cultura seqüencial de queratinócitos e melanócitose humanos sobre essa matriz contendo fibroblastos em seu interior.

## INTRODUCTION

The skin is the largest organ in the human body. In adults, its area ranges from 1.5 to 2.0 m^2^ and its weight from 8 to 10 kg. It contains around 1.0 × 10^11^ cells of epithelial, mesenchymal and neural origin and is composed of three distinct layers: the subcutaneous tissue (hypodermis), the dermis and the epidermis.^1^

The dermis is composed of dense collagen and elastic fibers produced by cutaneous fibroblasts, and the characteristics and distribution of these fibroblasts ensure the physical consistency of the skin (texture and elasticity).^2^ The epidermis contains keratinocytes close to the *stratum germinativum* (basal layer), and these ensure its renovation. It also contains melanocytes, the cells responsible for skin pigmentation and melanin synthesis. The melanin produced is progressively transferred to the keratinocytes.^3^

When a lesion occurs, the skin repairs itself through the proliferation and growth of the dermis (fibroblasts and other stromal cells) and/or epidermis (keratinocytes and melanocytes) cells that remain. In extensive and deep skin and mucosal lesions, destruction of the dermal and epidermal elements may occur. This makes the regeneration process slow and subject to complications. In such cases, autologous skin transplantation may be used, but its use is limited by the extent of the donor site and by the patient's clinical condition. Allotransplants from cadavers or volunteers are rejected after one or two weeks and provide only temporary coverage. Human skin or devitalized animal grafts, which may be either lyophilized or cooled in glycerol, accommodate the connective tissue and stimulate the growth of blood vessels, but usually become degraded at an early stage.^1^

Studies have been conducted with the aim of solving the problem of the need for large amounts of skin for transplantation in cases of burns and chronic skin ulcers, with scarce donor sites. These began by utilizing cells cultured *in vitro*, and experiments on both autologous^4,5^ and non-autologous^6^ cells were attempted. Models of human epidermis reconstructed *in vitro* were developed,^7,8^ but the clinical utilization of some of these models achieved only relative success. This was largely due to graft retraction problems caused by the absence of an adequate dermal bed (connective tissue)^9^ or the thinness of the grafts.^10^ More recently, the use of cells cultured *in vitro* in association with techniques to ensure adherence to the skin (fixing)^11-13^ has been studied. The use of dermal substitutes or equivalents containing collagen, in association with autologous cells cultured *in vitro*, has also been studied and has provided better results.^14-16^

The obtaining of skin containing associated dermis and epidermis, reconstructed with cells obtained from a small skin fragment, which is multiplied in the laboratory and usable in grafting, is still a theme to be explored. The utilization of acellular or collagen matrixes, for simultaneous growth of dermal and epidermal cells, has shown promising results.^17-19^

Collagen tissue used as matrix for cultured growth of fibroblasts (*in vitro*) provides a dermal equivalent with characteristics very similar to those found in human dermis, with good prospects of clinical application.^20,21^

The present study demonstrates a reproducible technique that can be rapidly implemented for obtaining human skin containing an association of dermis and epidermis, reconstructed *in vitro*. It is based on the culturing of fibroblasts inside a bovine collagen type I matrix, and sequential growth of keratinocytes and melanocytes on this collagen matrix.

## OBJECTIVE

To demonstrate a method for obtaining human skin reconstructed *in vitro* composed of associated dermis and epidermis, using fibroblasts cultivated in bovine collagen type I matrix and keratinocytes and melanocytes cultivated on this matrix.

## SETTING

Skin Cell Culture Laboratory of Faculdade de Ciências Médicas da Universidade Estadual de Campinas, Campinas, São Paulo, Brazil.

## METHODS

### Sample collection

Skin samples were collected from patients undergoing surgical procedures on the breast and abdomen at the Teaching Hospital of Universidade Estadual de Campinas, São Paulo, Brazil, by staff from the Discipline of Plastic Surgery. This procedure was approved by and was in conformity with the norms of the Ethics Committee of Faculdade de Ciências Médicas, Universidade Estadual de Campinas, and with the Declaration of Helsinki of 1975, as amended in 1983.

### Preparation of culture samples

Skin samples measuring 2.0 × 1.0 cm were collected and stored in sterile tempered glass jars, with preservation using 0.9% physiological serum at 4° C. All samples were processed within 12 hours of their collection.

The samples were separated from the adipose tissue and placed in jars with Hanks' Balanced Salt Solution (HBSS) (Gibco BRL, Grand Island, New York, United States, catalog no. 14025-092) plus antibiotic and antimycotic agents (sodium penicillin G, streptomycin and amphotericin B; Gibco catalog no. 15240-062). The whole skin samples were cut into smaller 1 to 2 mm fragments.

The liquid was removed from the jars, leaving behind the skin fragments, and 0.25% trypsin solution was added (Trypsin EDTA; Gibco catalog no. 25200-056). The jars were kept in an incubator at 37° C, with 5% CO_2_ tension, for 4 hours, which resulted in separation of the dermis from the epidermis.

The trypsin was then neutralized with fetal bovine serum (Gibco catalog no. 10437-028). The supernatant liquid was recovered from the jars and filtered using a 40 µm nylon filter (Falcon), and then centrifuged at 1200 rpm for 10 minutes.

The precipitate (cells) was washed with Hanks' Balanced Salt Solution plus antibiotic and antimycotic agents, and again centrifuged. Following this, one aliquot was removed for manual cell counting in a Neubauer chamber, using the Trypan Blue exclusion method.

### Cell culturing

After counting, the cells were distributed into culturing flasks (Corning), at a concentration of 1 × 10^5^ cells per cm^2^. They were then incubated at 37° C, with 5% CO_2_ tension, in specific culturing media for each cell type (keratinocytes, melanocytes and fibroblasts).

#### Culturing medium for keratinocytes:

Ke- ratinocyte culturing medium was used (Gibco catalog no. 10785-012), supplemented with L-glutamine 2 mM/ml, penicillin 100 UI/ml, streptomycin 0.1 mg/ml (Gibco catalog no. 10378-016) and fetal bovine serum 10%.

#### Culturing medium for melanocytes:

Melanocyte culturing medium MCDB 153 was used (Sigma Chemical Co., St. Louis, Missouri, United States, M7403), supplemented with L-glutamine 2 mM/ml, penicillin 100 UI/ml, streptomycin 0.1 mg/ml, fetal bovine serum 10%, bovine pituitary extract 50 µg/ml (Gibco catalog no. 13028-014), hydrocortisone 0.6 µg/ml (Sigma H0888) and bovine insulin 3 µg/ml (Gibco catalog no. 13007-018).

#### Culturing medium for fibroblasts:

Fibroblast culturing medium M199 was used (Gibco catalog no. 31100-035), supplemented with L-glutamine 2 mM/ml, penicillin 100 UI/ml, streptomycin 0.1 mg/ml and fetal bovine serum 10%.

For all the culturing media, the pH was maintained at around 7.8. The flasks were placed in an incubator at 37° C, with 5% CO tension. The culturing media were changed three times a week. The passage of cultured cells was performed when the cells were confluent.

### Cell passage

The culturing media in the flasks were removed. Trypsin 0.25% was added to each of the culturing flasks, which were placed in an incubator at 37° C, with 5% CO_2_ tension, for 10 minutes.

The trypsin was neutralized with fetal bovine serum, and the liquid containing the cells was transferred from the flasks to 50 ml Falcon tubes, for centrifugation at 1200 rpm for 10 minutes. The medium was removed and the cells resuspended at the desired concentration, now in the specific culturing medium. The cells were placed in the culturing flasks in an incubator, at 37° C, with 5% CO tension.

An average of 14 days was needed for obtaining sufficient number of cells (more than 2 × 10^6^), from each initial skin fragment measuring 2.0 × 1.0 cm. In about 21 days, a quantity of cells greater than 5 × 10^6^ was obtained.

These cells could be frozen in fetal bovine serum, with 10% dimethyl sulfoxide (DMSO; Sigma), in liquid nitrogen, and thus maintained for long periods. When they were thawed out, they presented viability of between 70 and 80%.

### Technique for obtaining human dermis reconstructed *in vitro*

Permeable and porous acellular bovine collagen type I matrix (Gen-col., block collagen, code 961.25, Genius line, Baumer Biomaterials Division, Mogi Mirim, São Paulo, Brazil), measuring approximately 10 × 15 × 25 mm, was utilized. A syringe was utilized to inject into this matrix a quantity of 2.0 × 10^6^ fibroblasts, in an M199 culture medium, supplemented with L-glutamine 2 mM/ml, penicillin 100 UI/ml, streptomycin 0.1 mg/ml and fetal bovine serum 10%.

The collagen type I matrix containing fibroblasts was placed on a Petri dish of approximately 10 cm^2^ and immersed in fibroblast culturing medium, supplemented with L-glutamine 2 mM/ml, penicillin 100 UI/ml, streptomycin 0.1 mg/ml and fetal bovine serum 10%. It was then placed in an incubator at 37° C, with 5% CO_2_ tension.

The culturing medium was changed three times a week. The human dermis reconstructed *in vitro* was obtained in seven days.

### Technique for obtaining human skin reconstructed *in vitro* containing associated dermis and epidermis

On the human dermis reconstructed *in vitro* on the Petri dish, a quantity of 5 × 10^6^ mixed epidermal cells were seeded at a melanocyte to keratinocyte ratio of 1:4 (i.e. approximately 1 × 10^6^ melanocytes and 4 × 10^6^ keratinocytes). The system was immersed in skin culturing medium composed of three parts of Iscove's Modified Dulbecco's Medium (IMDM) (Gibco catalog no. 12200036) plus one part of keratinocyte culturing medium, with 10% fetal bovine serum and Ca^2+^ 1.5 mM supplementation.

The culturing medium was changed three times a week. Human skin reconstructed *in vitro* containing associated dermis and epidermis was obtained in seven days (starting from the human dermis previously reconstructed *in vitro*).

### Control experiment

A quantity of 2.0 × 10^6^ fibroblasts in an M199 culture medium were placed on a Petri dish of approximately 10 cm^2^, and supplemented with L-glutamine 2 mM/ml, penicillin 100 UI/ml, streptomycin 0.1 mg/ml and fetal bovine serum 10%. The culturing medium was changed three times a week.

Seven days later, a quantity of 5 × 10^6^ mixed epidermal cells were seeded onto the Petri dish (melanocyte to keratinocyte ratio of 1:4). Skin culturing medium was added, composed of three parts IMDM plus one part keratinocyte culturing medium, with 10% fetal bovine serum and Ca^2+^ 1.5 mM supplementation. The culturing medium was changed three times a week.

After a further seven days, the skin obtained was removed from the culturing medium.

### Morphological studies on the human skin reconstructed *in vitro* containing associated dermis and epidermis and on the control experiment

Human skin samples containing associated dermis and epidermis reconstructed *in vitro* using bovine collagen type I matrix, and the skin specimens obtained in control experiments, were fixed in formaldehyde 10% and embedded in paraffin. Histological sections of 4 µm in thickness were prepared and stained with hematoxylineosin (HE), Masson's trichrome and PAS (periodic acid-Schiff).

## RESULTS

The experiment was successfully performed in triplicate, to validate the technique.

Macroscopically, both in the experiment to obtain human skin reconstructed *in vitro*, containing associated dermis and epidermis with utilization of bovine collagen type I, and in the control experiment, it was possible to observe the formation of a thin translucent pellicle. This was comparable, to the naked eye, to a very thin partial skin graft.

**Figure 1:**
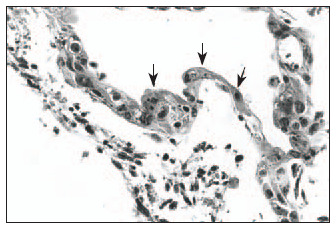
Epidermis in human skin reconstructed *in vitro*, with three to four keratinocyte layers, cuboid shaped at the base and flatter in the upper strata. Undulating surface, with a cuticular appearance (arrows). Clear distinction between dermis and epidermis (hematoxylin-eosin; original magnification 400 x).

**Figure 2:**
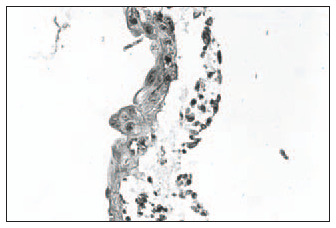
Clear dermal-epidermal limit in a sample of human skin reconstructed *in vitro*. Extracellular fibrillar matrix (bluish colored in histological sections) and fibroblasts in the dermis, organized in parallel to the epidermis (Masson's trichrome staining; original magnification 400 x).

**Figure 3:**
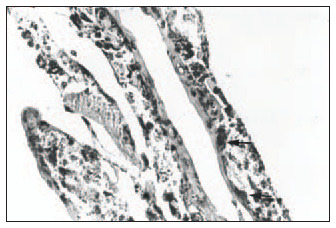
Presence of fuchsinophil material (glycogen, arrows) in the cytoplasm of keratinocytes, indicating cellular maturation of *in vitro* reconstructed human skin (periodic acid-Schiff; original magnification 400 x).

**Figure 4:**
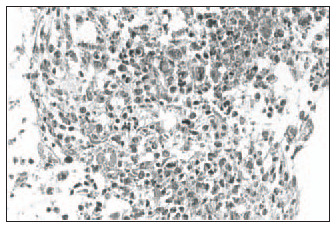
Presence of mixed dermal and epidermal cells, with predominance of fibroblasts. Absence of organoid architecture in human skin obtained in a control experiment of culturing (hematoxylin-eo-sin; original magnification 400 x).

**Figure 5:**
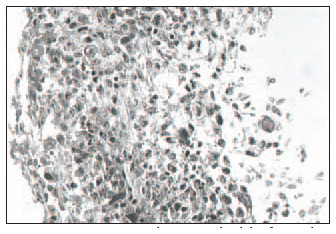
Dermis indistinguishable from the epidermis. No extracellular matrix observed in human skin obtained in a control experiment of culturing (Masson's trichrome staining; original magnification 400 x).

**Figure 6:**
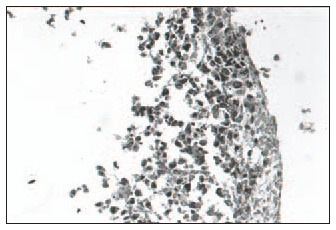
Absence of fuchsinophil material (glycogen) in the cytoplasm of keratinocytes in human skin obtained in a control experiment of culturing (periodic acid-Schiff; original magnification 400 x).

In the histological sections from the human skin reconstructed *in vitro* containing associated dermis and epidermis using collagen type I, it was possible to observe the following:

- Hematoxylin-eosin (HE): Clear distinction between dermis and epidermis. The epidermis was formed with three to four keratinocyte layers. These cells were cuboid at the base and flatter in the higher strata, with absence of the *stratum granulosum* and *stratum corneum*, and a wavy outer surface that had a cuticular appearance (Figure 1).
- Masson's trichrome: Clear dermal-epidermal border. Fibrillar extracellular matrix and fibroblasts in the dermis were organized in parallel to the epidermis (Figure 2).
- Periodic Acid-Schiff: Presence of fuchsinophil material (glycogen) in the cytoplasm of keratinocytes (Figure 3).
  In the histological sections from the skin obtained in the control experiment, the following was observed:
- Hematoxylin-eosin (HE): Presence of mixed dermal and epidermal cells, with predominance of fibroblasts. Absence of organoid architecture (Figure 4).
- Masson's trichrome: The dermis was not distinguishable from the epidermis. The extracellular matrix was not observed (Figure 5).
- Periodic Acid- Schiff: No fuchsinophil material (glycogen) was observed in the cytoplasm of keratinocytes (Figure 6).

## DISCUSSION

With the utilization of a bovine collagen type I matrix for fibroblast growth, in a selective culturing medium, a human dermis reconstructed *in vitro* that was similar to normal human dermis was obtained, with its cells (fibroblasts) organized in parallel within a three-dimensional structure (collagen).

On this human dermis reconstructed *in vitro*, a clearly differentiated human epidermis reconstructed *in vitro* was reproduced by keratinocyte and melanocyte culturing in a skin-adequate medium.

Therefore, through sequential culturing of dermal cells (fibroblasts) in a bovine collagen type I matrix, and of epidermal cells (keratinocytes and melanocytes) on this matrix, a human skin composed of associated dermis and epidermis reconstructed *in vitro* can be obtained. This reconstructed skin presents collagen in the dermis, and has fibroblasts, keratinocytes and melanocytes positioned in a manner analogous to the equivalent human skin, while maintaining the due thickness proportions in relation to human skin *in vivo*. The stratification of the epidermis in the human skin that was reconstructed *in vitro* containing associated dermis and epidermis has characteristics similar to those found *in vivo*. Human skin reconstructed *in vitro* containing associated dermis and epidermis was obtained in 14 days, starting from sufficient numbers of dermal cells (fibroblasts) and epidermal cells (melanocytes and keratinocytes).

In histological sections, the human skin reconstructed *in vitro* obtained in the control experiment presented dermal cells (fibroblasts) and epidermal cells (keratinocytes and melanocytes) without any architectural organization, and without any evidence of constituted dermis and epidermis. The failure to obtain human skin containing distinguishable dermis and epidermis through reconstruction *in vitro* in the control experiment confirms the importance of the utilization of a bovine collagen type I matrix to obtain a human skin reconstructed *in vitro* that is equivalent to human skin *in vivo*.

Collagen is available in abundance and can be easily purified from living organisms. It is biodegradable, bioabsorbable and nontoxic, and has low antigenicity and excellent biocompatibility, in comparison with other natural polymers like albumin and gelatin. Among the main applications of collagen in the field of medicine are its uses in ophthalmological prostheses and as sponges (biological dressings) for burns and skin ulcers. In tissue engineering, it is utilized as the basic matrix for the development of cell systems and in constructing artificial substitutes for vessels and valves.^22^

One of the few disadvantages of pure collagen type I is that it has a relatively high cost. However, the high cost of treating skin lesions using this when grafting donor sites are scarce is perfectly justifiable because of the decreased treatment duration,^23,24^ and also the better survival rates and the quality of the end result.^25^ Clinical reactions to collagen are rare, but two cases of allergy to bovine collagen, mediated by immunoglobulin E (IgE), have been reported. In both of these cases, the patients developed conjunctival edema as a reaction to the topical application of highly purified bovine collagen to their eyes, during ophthalmological surgery.^26^

The utilization of a bovine collagen type I membrane on an area of skin loss may lead to the formation of neodermis, through migration of the patient's own fibroblasts into the membrane, followed by cell multiplication and maturation. Spontaneous epithelization may take place over the neodermis, through epithelial growth occurring in the lateral region of the wound, or epithelization may be induced by placing autologous keratinocytes cultured *in vitro* on the neodermis.^14^ The possibility of clinical utilization of autologous human skin containing associated dermis and epidermis that is completely reconstructed *in vitro* is much more advantageous than the methods employed previously.

In the present study, skin samples measuring 2.0 × 1.0 cm were removed from the patients. In about two weeks, we had quantities of fibroblasts greater than 2 × 10^6^. In three weeks, we had quantities of fibroblasts greater than 5 × 10^6^ each, and melanocyte and keratinocyte quantities of around 5 × 10^6^ each. In four weeks, we had keratinocytes and melanocytes in a number higher than 20 × 10^6^. We needed 2 × 10^6^ fibroblasts and 5 × 10^6^ kera- tinocytes and melanocytes in a ratio of 4:1 (4 × 10^6^ keratinocytes and 1 × 10^6^ melanocytes), in order to obtain human skin fragments reconstructed *in vitro* measuring 2.0 × 2.0 cm after two weeks of culturing. Therefore, the method here presented offers the possibility of obtaining human skin, containing dermis and epidermis reconstructed *in vitro*, of twice the size of the original skin removed from the patient, by means of conventional cell culturing over a four-week period. The utilization of cell culturing acceleration methods^27^ may supply sufficient numbers of cells for seeding inside the bovine collagen type I matrix in a shorter time, with a concomitant decrease in the time needed for obtaining human skin reconstructed *in vitro*.

The freezing of cells with maintenance of their viability for long periods offers a way of obtaining cells for culturing and consequent reconstitution of human skin containing autologous dermis and epidermis *in vitro*, in cases in which there is initial failure in the clinical treatment of skin ulcers and burns, without the need for new skin samples. Dimethyl sulfoxide is added to the fetal bovine serum at a concentration of 10% during the freezing process, to avoid cell crystallization.

While the epidermis is forming in the skin-culturing medium, on the bovine collagen type I matrix, it is essential to add Ca^2+^ at a concentration of 1.5 mM to the medium, so that the formation of a pellicle occurs at the location where the *stratum corneum* would be (Figure 1). This was demonstrated in previous studies that aimed at obtaining human epidermis reconstructed *in vitro*.^8,28^ The pellicle formed during the process for obtaining human skin reconstructed *in vitro* composed of associated dermis and epidermis, where the *stratum corneum* would be located, is extremely thin. However, it may thicken if it is exposed to air (maintained at the air-liquid interface), thus forming a real *stratum corneum*.

This model for human skin reconstructed *in vitro*, containing associated dermis and epidermis, offers an excellent investigative system, particularly in relation to efficiency and toxicology tests on new chemical products and drugs *in vitro*. It has the advantage of presenting dermal components, in comparison with systems presented in studies that made use of the epidermis alone.^28,29^

The present model is not expected to enable research on the immunological effects of reactions mediated by Langerhans cells or by ultraviolet-induced macrophages, as demonstrated in studies that made use of epidermis reconstructed *in vitro* separately.^30^ However, this could be perfectly achieved by cultivating Langerhans cells separately,^31^ and introducing these cells together with keratinocytes and melanocytes seeded on the human dermis reconstructed *in vitro*, thus obtaining human skin reconstructed *in vitro* with immune function.

This model may also allow research on the physiopathology and possible therapeutic options for pigmentation disorders such as vitiligo, melasma and melanocytic nevus, for which the origin is still indeterminate. Several other models for human skin reconstructed *in vitro*, with a variety of laboratory, experimental and clinical applications, are expected to emerge and undergo investigation over the coming years.

We consider that the present model for human skin reconstructed *in vitro*, containing associated dermis and epidermis, is compatible with and similar to human skin *in vivo*. It presents advantages over previous models for artificial skin substitutes^21,32-34^ and epidermis reconstructed *in* vitro.^10,11,13,35^

## CONCLUSIONS

It is possible to obtain sufficient numbers of cells from human fibroblast culturing that can be injected into a bovine collagen type I matrix, thereby enabling the formation of a human dermis reconstructed *in vitro*. Following this, through human keratinocyte and melanocyte culturing on this human dermis reconstructed *in vitro*, it is possible to obtain a completely differentiated human skin reconstructed *in vitro*, composed of associated dermis and epidermis.

We believe that the present model for human skin reconstructed *in vitro* has excellent applicability in relation to laboratory studies and good prospects for clinical use. Therefore, our next step would be to characterize this model for human skin reconstructed *in vitro* composed of associated dermis and epidermis, by means of immunohistochemical and molecular studies, with the aim of using it for treating burns and chronic skin ulcers.
